# Supplementary material for: Alpha-Gal and Cross-Reactive Carbohydrate Determinants in the N-Glycans of Salivary Glands in the Lone Star Tick, Amblyomma americanum
Source: Vaccines (Basel). 2020 Jan 9;8(1):18. doi: 10.3390/vaccines8010018 (PMC7157712; doi:10.3390/vaccines8010018)

SFig. 1

N-glycan profiles in the lone star tick. Samples are for females and males of salivary glands (FSG and MSG) and carcasses (MCC and FCC) before the onset of feeding, 2 days after, and 5 days after feeding attachments (0dAF, 2dAF, and 5dAF). The percent total ion is shown with the heatmap with m indicating the mix of two different glycans. The m/z and deduced glycan structures are shown.

			FSG-UF	FSG-2dF	FSG-5dF	MSG-UF	MSG-2dF	MSG-5dF	MCC-5dF	FCC-5dF
733.3734	(HexNAc)$_2$ (Deoxyhexose)$_1$	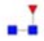	0	0	0	0	0	0	0	6
763.3841	(Hex)$_1$ (HexNAc)$_2$		0	0	0	0	0	0	0	5
937.4541	(Hex)$_1$ (HexNAc)$_2$ (Deoxyhexose)$_1$	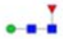	5	3.04	0	0	0.82	15.65	32	10
967.4837	(Hex)$_2$ (HexNAc)$_2$		0	8.18	0	11	3.8	4.85	15	71
1111.563	(Hex)$_1$ (HexNAc)$_2$ (Deoxyhexose)$_2$	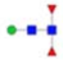	0	0	0	0	0	0	11	0
1141.573	(Hex)$_2$ (HexNAc)$_2$ (Deoxyhexose)$_1$	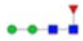	0	1.24	3.39	3	0.08	0.82	21	5
1171.584	(Hex)$_3$ (HexNAc)$_2$	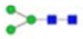	0	3.43	1.13	0	0.53	0.01	4	1
1315.662	(Hex)$_2$ (HexNAc)$_2$ (Deoxyhexose)$_2$	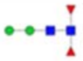	0	0	0	0	0	0	5	0
1331.658	(Hex)$_3$ (HexNAc)$_2$ (Pent)$_1$	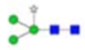	0	0	0.03	0	0	0.07	0	0
1345.672	(Hex)$_3$ (HexNAc)$_2$ (Deoxyhexose)$_1$	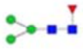	0	1.32	2.16	0	0.22	0.04	7	0
1375.684	(Hex)$_4$ (HexNAc)$_2$	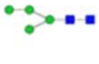	0	0.18	0.03	0	0.01	0	0	0
1386.7	(Hex)$_2$ (HexNAc)$_3$ (Deoxyhexose)$_1$	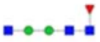	0	0	0.01	0	0	0	0	0
1519.762	(Hex)$_3$ (HexNAc)$_2$ (Deoxyhexose)$_2$	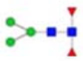	0	0	0	0	0	0	1	0
1549.772	(Hex)4 (HexNAc)2 (Deoxyhexose)1	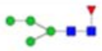	0	0.02	0	15	0.01	0	1	2
1579.787	(Hex)$_2$ + (Man)$_3$(GlcNAc)$_2$	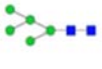	5	12.92	0.58	20	19.36	12.01	0	0
1590.8	(HexNAc)$_1$ (Deoxyhexose)$_1$ + (Man)$_3$(GlcNAc)$_2$	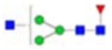	0	0.23	1.13	0	0.01	0.94	0	0

1661.836	$(HexNAc)_2$ + $(Man)_3(GlcNAc)_2$	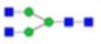	0	0	2.64	0	0	0	0	0
1753.872	$(Hex)_2$ $(Deoxyhexose)_1$ + $(Man)_3(GlcNAc)_2$	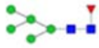	0	6.15	0	2	7.13	0	0	0
1783.882	$(Hex)_3$ + $(Man)_3(GlcNAc)_2$	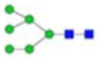	17	12.21	1.46	14	13.66	7.86	0	0
1794.899	$Hex)_1$ $(HexNAc)_1$ $(Deoxyhexose)_1$ + $(Man)_3(GlcNAc)_2$	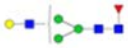	0	0	0.72	0	0.61	0	0	0
1824.909	$(Hex)_2$ $(HexNAc)_1$ + $(Man)_3(GlcNAc)_2$	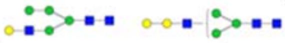	0	0	0	0	0	0.03m	0	0
1835.925	$(HexNAc)_2$ $(Deoxyhexose)_1$ + $(Man)_3(GlcNAc)_2$	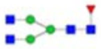	0	0	3.36	0	0	0.79	1	0
1865.936	$(Hex)_1$ $(HexNAc)_2$ + $(Man)_3(GlcNAc)_2$	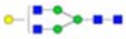	0	0	1.39	0	0	0	0	0
1984.983	$(Hex)_2$ $(HexNAc)_1$ $(Pent)_1$ + $(Man)_3(GlcNAc)_2$	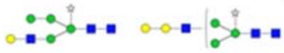	0	0	0	0	0	1.06m	0	0
1987.982	$(Hex)_4$ + $(Man)_3(GlcNAc)_2$	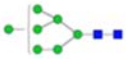	15	7.35	0.69	9	8.41	3.16	0	0
1999.006	$(Hex)_2$ $(HexNAc)_1$ $(Deoxyhexose)_1$ + $(Man)_3(GlcNAc)_2$	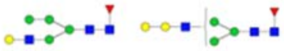	0	0.75m	1.13m	0	0	0	0	0
2029.01	$(Hex)_3$ $(HexNAc)_1$ + $(Man)_3(GlcNAc)_2$	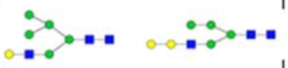	0	0	0.04m	0	0	0	0	0
2040.02	$(Hex)_1$ $(HexNAc)_2$ $(Deoxyhexose)_1$ + $(Man)_3(GlcNAc)_2$	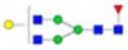	2	0	3.86	0	0.53	7.11	0	0
2067.037	$(HexNAc)_3$ $(Pent)_1$ + $(Man)_3(GlcNAc)_2$	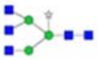	8	0	3.33	0	0	12.67	0	0
2070.036	$(Hex)_2$ $(HexNAc)_2$ + $(Man)_3(GlcNAc)_2$	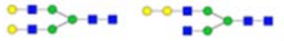	0	0	1.33m	1	0	0	0	0
2081.051	$(HexNAc)_3$ $(Deoxyhexose)_1$ + $(Man)_3(GlcNAc)_2$	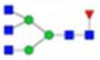	2	6.58	38.82	1	5.39	12.65	1	0
2111.067	$(Hex)_1$ $(HexNAc)_3$ + $(Man)_3(GlcNAc)_2$	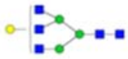	0	0	2.92	0	0.97	0	0	0
2192.083	$(Hex)_5$ + $(Man)_3(GlcNAc)_2$	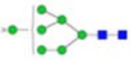	6	4.56	0.32	4	4.38	2.4	0	0
2203.09	$(Hex)_3$ $(HexNAc)_1$ $(Deoxyhexose)_1$ + $(Man)_3(GlcNAc)_2$	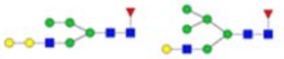	0	0.01	0.39m	0	0.01m	0.25m	0	0
2230.107	$(Hex)_2$ $(HexNAc)_2$ $(Pent)_1$ + $(Man)_3(GlcNAc)_2$	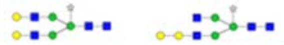	0	0	0.34m	0	0	0	0	0
2233.11	$(Hex)_4$ $(HexNAc)_1$ + $(Man)_3(GlcNAc)_2$	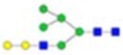	0	0.01	0.04	0	0.01	0.04	0	0

2244.119	Hex)$_2$ (HexNAc)$_2$ (Deoxyhexose)$_1$ + (Man)$_3$(GlcNAc)$_2$	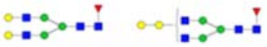	7	2.15m	2.87m	2	3.09	4.08m	0	0
2271.135	(Hex)$_1$ (HexNAc)$_3$ (Pent)$_1$ + (Man)$_3$(GlcNAc)$_2$	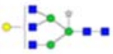	0	0	1.33	0	0	0	0	0
2285.151	(Hex)$_1$ (HexNAc)$_3$ (Deoxyhexose)$_1$ + (Man)$_3$(GlcNAc)$_2$	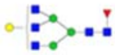	18	6.65	14.09	9	5.01	2.67	0	0
2315.162	(Hex)$_2$ (HexNAc)$_3$ + (Man)$_3$(GlcNAc)$_2$	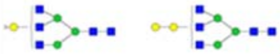	0	0	0.75m	0	0.55	0	0	0
2326.178	(HexNAc)$_4$ (Deoxyhexose)$_1$ + (Man)$_3$(GlcNAc)$_2$	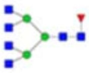	0	0	0.69	0	0	0	0	0
2396.183	(Hex)6 + (Man)3(GlcNAc)2	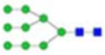	2	5.32	0.31	2	4.75	3.07	0	0
2448.227	(Hex)$_3$ (HexNAc)$_2$ (Deoxyhexose)$_1$ + (Man)$_3$(GlcNAc)$_2$	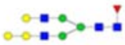	0	0	0.51	0	0.01	1.74	0	0
2448.222	(Hex)3 (HexNAc)2 (Deoxyhexose)1 + (Man)3(GlcNAc)2	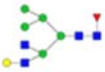	0	0	0	1	0	0	0	0
2475.24	(Hex)$_2$ (HexNAc)$_3$ (Pent)$_1$ + (Man)$_3$(GlcNAc)$_2$	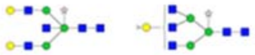	0	0.96m	0.11	0	0	1.19	0	0
2478.24	(Hex)$_4$ (HexNAc)$_2$ + (Man)$_3$(GlcNAc)$_2$	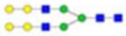	0	0.09	0.26	0	0	0	0	0
2489.252	(Hex)$_2$ (HexNAc)$_3$ (Deoxyhexose)$_1$ + (Man)$_3$(GlcNAc)$_2$	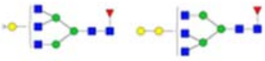	13	14.29	2.82m	6	14.76	1.34m	0	0
2652.322	(Hex)$_4$ (HexNAc)$_2$ (Deoxyhexose)$_1$ + (Man)$_3$(GlcNAc)$_2$	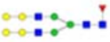	0	0.01	1.59	0	0.01	1.41	0	0
2693.352	(Hex)$_3$ (HexNAc)$_3$ (Deoxyhexose)$_1$ + (Man)$_3$(GlcNAc)$_2$	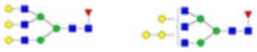	0	0.9	0.61m	0	1.77	0.64	0	0
2883.436	(Hex)$_4$ (HexNAc)$_3$ (Pent)$_1$ + (Man)$_3$(GlcNAc)$_2$	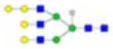	0	0	0	0	0	0.69	0	0
2897.451	(Hex)$_4$ (HexNAc)$_3$ (Deoxyhexose)$_1$ + (Man)$_3$(GlcNAc)$_2$	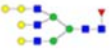	0	0	0	0	0	0.85	0	0
2897.451	(Hex)$_4$ (HexNAc)$_3$ (Deoxyhexose)$_1$ + (Man)$_3$(GlcNAc)$_2$	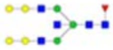	0	1.49	2.81	0	0.6	0	0	0
2938.478	(Hex)$_3$ (HexNAc)$_4$ (Deoxyhexose)$_1$ + (Man)$_3$(GlcNAc)$_2$	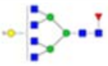	0	0	0	0	0.34	0	0	0
3142.578	(Hex)$_4$ (HexNAc)$_4$ (Deoxyhexose)$_1$ + (Man)$_3$(GlcNAc)$_2$	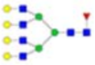	0	0	0	0	0.08	0	0	0
3602.783	(Hex)$_3$ (HexNAc)$_3$ (NeuAc)$_3$ + (Man)$_3$(GlcNAc)$_2$	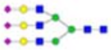	0	0	0	0	0.16	0	0	0
3604.799	(Hex)$_4$ (HexNAc)$_5$ (NeuGc)$_1$ + (Man)$_3$(GlcNAc)$_2$	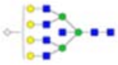	0	0	0	0	0.78	0	0	0

SFig.2

Supplementary data 2. Mass spectrometry spectra for the salivary glands of female fed for two days (F_SG_2dAF).

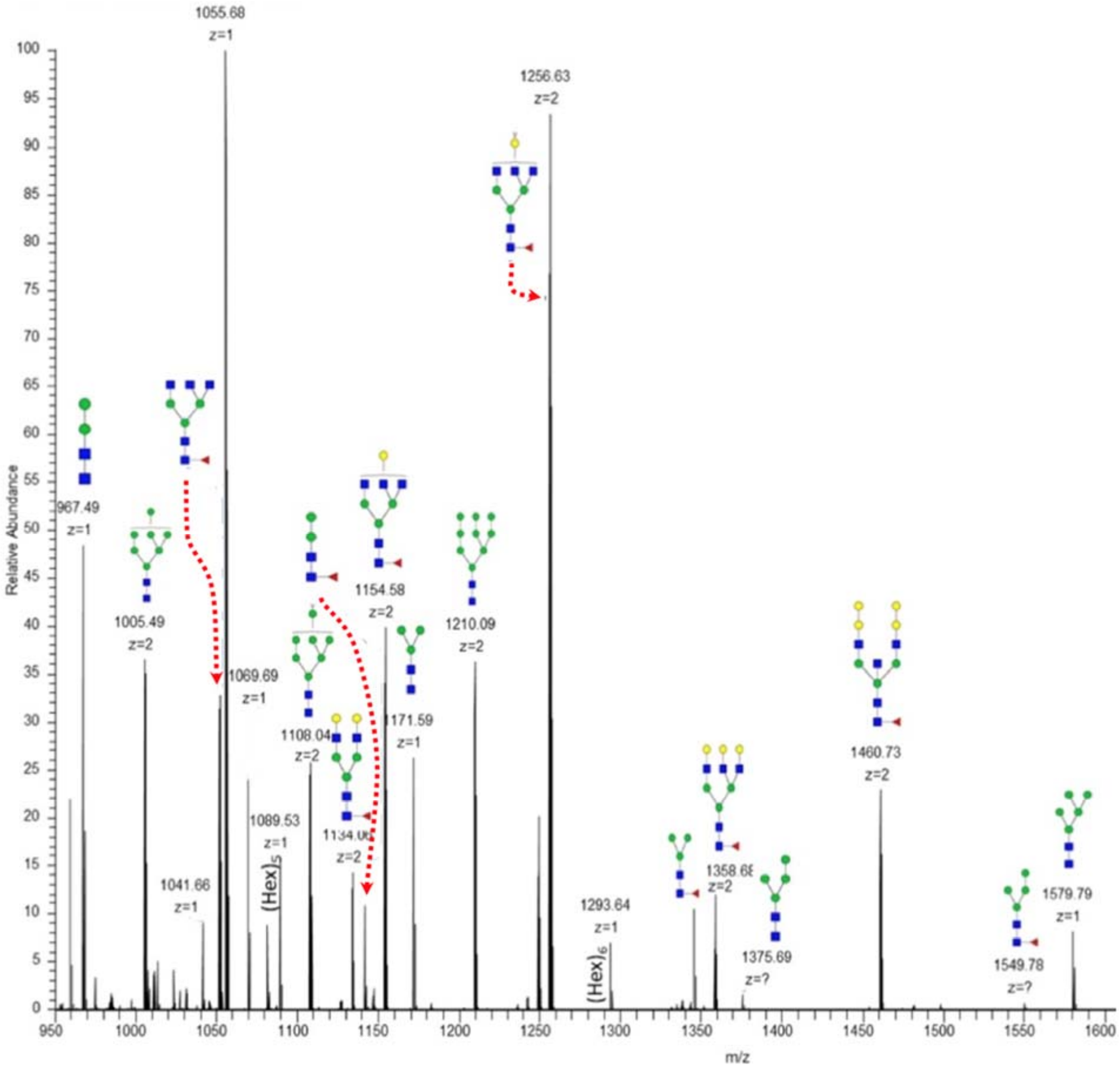

SFig.3
Supplementary data 3. Mass spectrometry spectra for the salivary glands of female fed for five days (F_SG_5dAF).

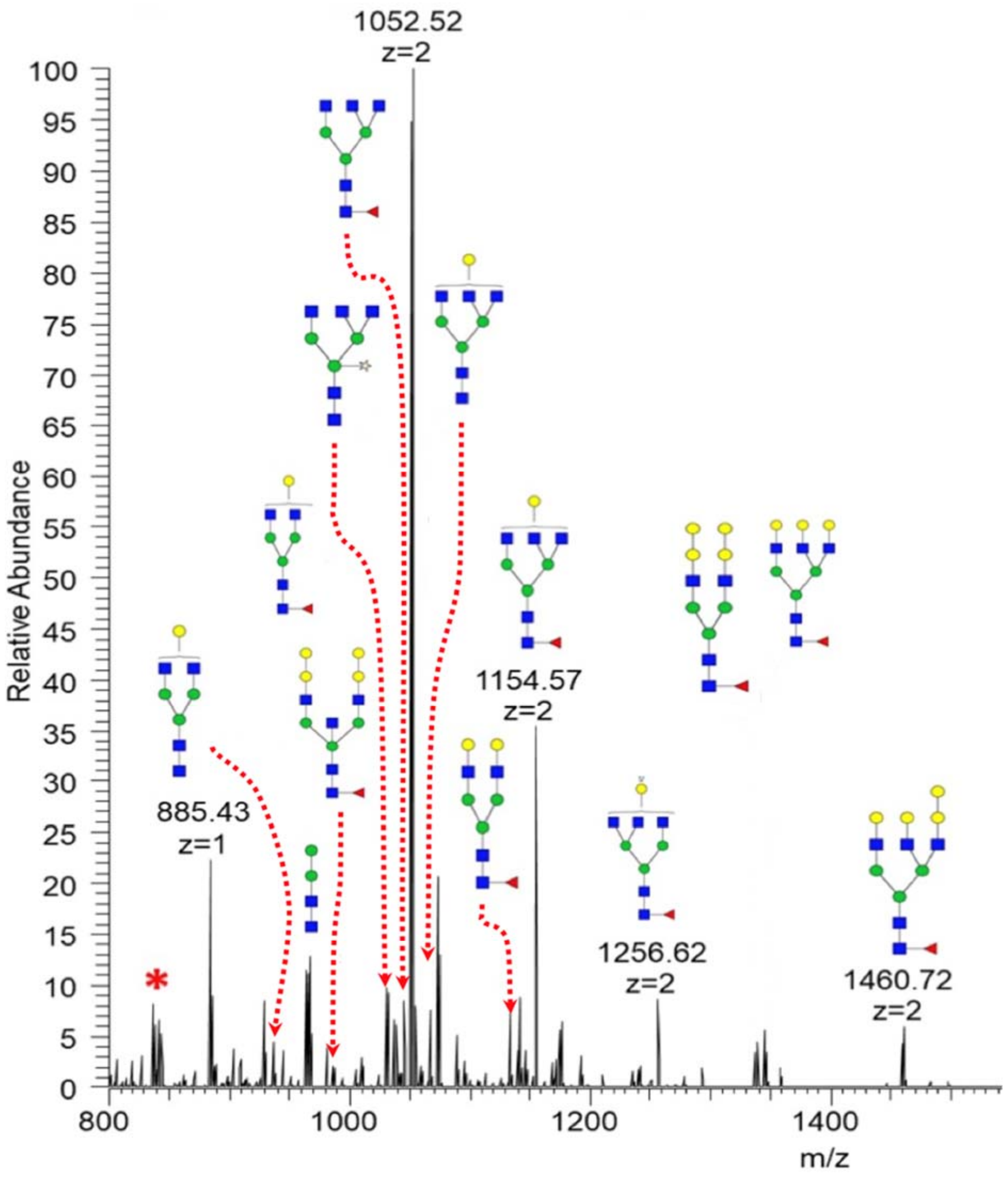

SFig.4
Supplementary data 4. MS/MS spectra of a Xylose-containing glycoform found in female salivary gland after 5 day feeding (F_SG_5dAF).

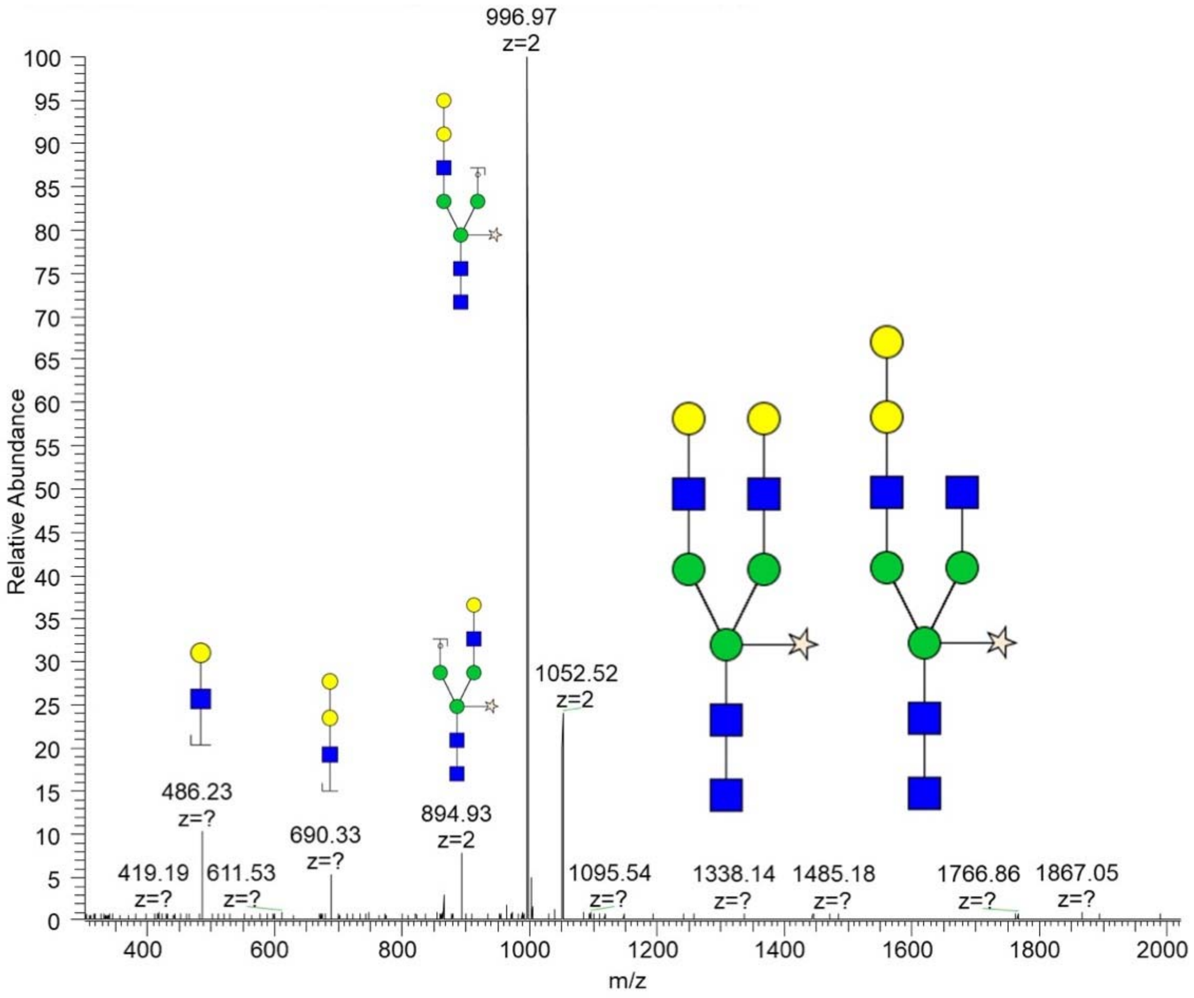

SFig.5

Supplementary data 5. MS/MS spectra of a mono- and di- fucose (m/z 1345) found in female carcass after 5 day feeding. Note that the decay pattern supports the 1,3 fucose paucimannose.

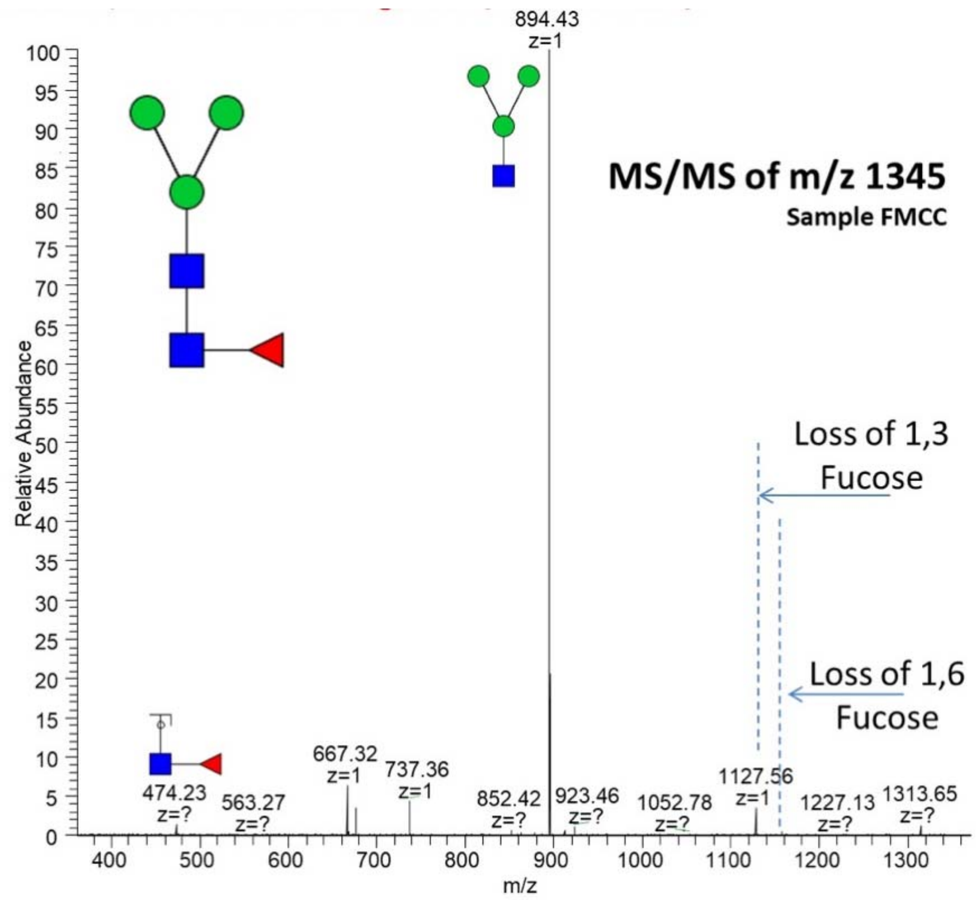

Supplement: Supplementary file 1 [file vaccines-08-00018-s001.pdf]
